# Neural Correlates of Distorted Self-concept in Individuals With Internet Gaming Disorder: A Functional MRI Study

**DOI:** 10.3389/fpsyt.2018.00330

**Published:** 2018-07-25

**Authors:** Min-Kyeong Kim, Young Hoon Jung, Sunghyon Kyeong, Yu-Bin Shin, Eunjoo Kim, Jae-Jin Kim

**Affiliations:** ^1^Department of Psychiatry, Yonsei University College of Medicine, Seoul, South Korea; ^2^Institute of Behavioral Science in Medicine, Yonsei University College of Medicine, Seoul, South Korea; ^3^Brain Korea 21 PLUS Project for Medical Science, Yonsei University, Seoul, South Korea

**Keywords:** internet gaming disorder, self-discrepancy, actual self-concept, ideal self-guide, inferior parietal lobule

## Abstract

**Background and aims:** Discrepancy between ideal self-guide and actual self-concept evoke dejection-related feeling, and often individuals with internet gaming disorder (IGD) use games as the tool to escape those negative emotions. The aim of this study was to evaluate the pattern of self-discrepancy based on actual and ideal self-images and elucidate the neural correlates underlying the distorted self in individuals with IGD.

**Methods:** Nineteen male individuals with IGD and 20 healthy controls (HCs) underwent functional magnetic resonance imaging where they decided on whether they agreed with the adjectives describing their actual or ideal self on a four-point Likert Scale. Two-sample *t*-test on the self-discrepancy contrast was conducted for neuroimaging analysis and correlation analysis was performed between the behavioral data and regional activities.

**Results:** The IGD group evaluated both their ideal self and actual self more negatively than the HC group. Actual self-concept was associated with satisfaction with psychological needs as opposed to ideal self-guide. Brain activity in the inferior parietal lobule was significantly decreased in individuals with IGD relative to HCs in the self-discrepancy contrast. In addition, neural activity during evaluating actual self-concept showed a significant group difference.

**Conclusion:** These results provide novel evidence for distorted self-concept of people with IGD. Individuals with IGD had a negative ideal and actual self-image. Neurobiologically, dysfunction in the inferior parietal lobule associated with emotional regulation and negative self-evaluation was found in IGD. Considering the characteristics of IGD that often develop in adolescence, this self-concept problem should be noted and applied with appropriate therapy.

## Introduction

Internet gaming disorder (IGD) is characterized by functional impairment in personal or social life from excessive internet game use. It is an emerging disorder due to the spread of the Internet (1). This condition has a significant symptomatic similarity to substance use disorders and behavior addiction (2, 3). However, the difference between other addictive mediators and Internet games is that games are relatively easy to access even at a younger age (4). Thus, it is no surprise that IGD mainly occurs in teenagers (5). One of the developmental tasks to be accomplished in adolescence is the formation of identity (6). Because games reduce other interests in daily life, adolescents preoccupied with games might be thwarted in achieving the formation of identity and other developmental tasks (7).

Self-discrepancy theory (SDT) explains that distorted self-images can cause various emotional discomfort (8). This theory assumes three domains of self: actual self, ideal self, and ought self. Actual self-concept is the perception of one's own attributes, ideal self-guide is the representation of the attributes that the person wants to possess, and ought self-guide is the representation of the properties that someone else believes the person should possess. Negative emotions arise when there is high discrepancy between the domains. Specifically, a significant mismatch between actual self-concept and ideal self-guide is related to dejection such as low self-esteem or frustration (8–11). Because Internet games can be used as a means of escaping these negative emotions, it is important to understand the relationship between IGD and self-discrepancy (12–14).

SDT has been used to explain several psychiatric disorders including addictive disorders. Studies show that substance abusers show a high level of self-discrepancy (15) and that distress associated with self-discrepancy predicts alcohol consumption (16). Among addictive disorders, distorted self-image or self-discrepancy in IGD may be clinically more important as IGD-related symptoms occur at a young age. Game users could become confused about their identity as they are constantly exposed to avatars similar to their ideal fantasy (17–19). Despite concerns about identity confusion, little is known about which specific domains of self-images are associated with self-discrepancy.

Impairment of self-regulation is one of the major psychopathologies of addiction (20). Self-regulation ability is related to how well basic psychological needs are satisfied (21, 22). These basic psychological needs, consisting of autonomy, competence, and relatedness, are important factors affecting individual growth and integration (22–24). If these are not satisfied from a young age, individuals may struggle to form a stable self-image. It is known that people who are dissatisfied with basic psychological needs use social media networks (25), as well as Internet games (26). Despite the connection between basic psychological needs and self-image, the relationship between the two has not been elucidated.

The concept of self-discrepancy is mostly studied observationally using self-report to support the theory, and little is known about the neural correlates of self-discrepancy. A single study suggests that self-discrepancy was associated with activation in the reward system including the striatum, which might be linked to the desire for one's ideal self (27). In terms of self-referential processing, which is the basis of self-discrepancy, the medial prefrontal cortex (MPFC) is involved (28, 29). Also, a meta-analysis showed that individuals with IGD have the prefrontal dysfunction related to their self-regulation problem (30). Given the importance of self-image in adolescence, investigating the neurobiological underpinnings of self-discrepancy in IGD would play an important role in understanding the psychopathology and establishing treatment strategies of the disorder.

The aim of this study was to investigate the neural correlates underlying the distorted self of individuals with IGD in relation to their satisfaction with basic psychological needs. We developed a self-concept task for fMRI to evaluate the attitudes of self-discrepancy based on actual and ideal self-images. Considering previous research that games are used to avoid negative emotions caused by self-discrepancy, we hypothesized that individuals with IGD would show higher self-discrepancy. Also, individuals with IGD who were frequently exposed to game avatars that were close to their ideal fantasy would have impairment in both actual self-concept and ideal self-guide. Neurobiologically, we hypothesized that individuals with IGD would show dysfunction in the striatum and MPFC, which are associated with self-discrepancy.

## Methods

### Participants

In total, 19 individuals with IGD (mean age ± standard deviation: 23.3 ± 2.4) and 20 age-matched healthy controls (HCs) (mean age ± standard deviation: 23.4 ± 1.2) participated in this study. Considering the epidemiology of IGD (31–33), male participants in their 20 s playing internet games more than 30 h a week were recruited through internet advertisement. Then, participants who met the DSM-5 proposed criteria for IGD (1) in a psychiatric interview were enrolled. Participants with IGD who had a history of depressive disorder or attention deficit hyperactivity disorder were included in consideration of various comorbid conditions (1). Considering that the features of IGD have not yet been fully studied, however, participants who were suffering from on-going psychiatric illness except IGD or those who suffered from other addictive disorders were excluded. All participants were right-handed (34) and did not have medical and neurological illness. This study was approved by the Institutional Review Board of Yonsei University Gangnam Severance Hospital and carried out in accordance with the Declaration of Helsinki. Written informed consent was obtained from all participants before the study began.

### Assessment scale

To measure the presence and the severity of Internet dependency, the internet addiction test (IAT) was used (35). The IAT is a 20-item scale with a 5-point score, ranging from 1 (very rarely) to 5 (very frequently). Scores higher than 50 indicate problematic internet use. Participants were instructed to evaluate their internet use, especially on the basis of internet game use. Degree of psychological needs satisfaction was assessed by the Basic Psychological Needs Scale (BPNS) (36, 37). This consisted of 21 items with 7-point Likert scale (1: not at all true to 7: very true). Higher scores meant a higher level of psychological needs satisfaction.

### Behavioral task

Participants performed the self-concept task during fMRI scanning. The task asked the participants' view of actual and ideal self. A sentence describing actual self (e.g., I am a modest person) and ideal self (e.g., I want to be a modest person) was presented on the screen and participants answered how well the sentence described themselves by clicking one of four buttons (1: strongly disagree to 4: strongly agree). A total of 48 trait adjectives (24 positive and 24 negative) were used in these sentences. The task comprised 8 blocks for each condition (actual self and ideal self). A block lasted for 32 s and a 16 s rest period was placed between the blocks. In each block, 6 different sentences (3 sentences with a positive adjective and 3 sentences with a negative adjective) were presented for 3 s each with an inter-stimulus interval jittered between 0.5 and 3.5 s. The sequence of experimental blocks and sentences was pseudo-randomized.

### Image acquisition

MRI data were acquired on a 3 Tesla scanner (Magnetom Verio, Siemens Medical Solutions, Erlangen, Germany). Functional images were collected using a gradient echo planar imaging sequence (echo time = 30 ms, repetition time = 2,000 ms, flip angle = 90°, slice thickness = 3 mm, number of slices = 30, and matrix size 64 × 64). Three scans were discarded before image acquisition started. Structural images were also collected using a 3D spoiled-gradient-recall sequence (echo time = 2.46 ms, repetition time = 1,900 ms, flip angle = 9°, slice thickness = 1 mm, number of slices = 176, and matrix size = 256 × 256).

### Behavioral data analysis

A “positivity score” was calculated as the average of 48 responses per condition indicating the positive level of the actual and the ideal self. Higher scores indicated that the participants had a more positive representation of themselves. Also, a “self-discrepancy score” was constructed by subtracting the positivity score of the ideal self from that of the actual self. Analysis of variance (ANOVA) was performed to evaluate the main and interaction effect of group (HC vs. IGD) and condition (actual self vs. ideal self) on the positivity scores. In addition, independent *t*-test was used for group comparison of the self-related scores (positivity scores and self-discrepancy scores), and Pearson's correlation analysis was performed between these scores and BPNS scores in each group. SPSS (ver. 23; SPSS Inc., Chicago, IL, USA) was used and a *p*-value < 0.05 was considered significant.

### Neuroimaging data analysis

Preprocessing and analysis of fMRI data were performed with Statistical Parametric Mapping, version 12 (Wellcome Department of Cognitive Neurology, University College London). fMRI images were corrected for the differences of slice acquisition time. Then, individual head motions were corrected based on realignment on the first image. Functional images were co-registered on the structural images. The structural images were normalized to the standard template spatially, and transformation matrices were applied to the functional images. These images were smoothed with a Gaussian kernel of 6 mm full-width at half-maximum.

For the individual analysis, the actual self and ideal self conditions convolving the canonical hemodynamic response function were used as regressors of interest and 6 movement parameters were included as regressors of non-interest in general linear model. Three main contrast images were created: actual self, ideal self, and self-discrepancy (ideal self**—**actual self). One sample *t*-test for the comparison between the actual self and ideal self was performed in each group. Full factorial analysis of variance was applied to investigate the interaction effect between group and condition, and additional two sample *t*-test was performed on self-discrepancy contrast images. Results were considered significant at a threshold of corrected *p* < 0.05, which corresponded to the family-wise error corrected significance at the cluster level with a cluster-defining threshold of *p* < 0.005. For a *post-hoc* analysis, whole clusters identified in two-sample *t*-test were defined as the regions of interest (ROIs) and their regional activity was extracted with MarsBaR version 0.44. Using SPSS, Pearson's correlation analysis was performed between neural activities in each contrast and behavioral data (BPNS scores and self-discrepancy score). Also, regional activities for the actual self and ideal self conditions were compared using independent *t*-tests. Results were considered significant at *p* < 0.05.

## Results

### Clinical characteristics and behavioral response to the self-concept task

Demographic and clinical characteristics are presented in Table 1. Scores of IAT (IGD: 73.0 ± 9.7, HC: 24.9 ± 6.1, *t* = 18.4, *p* < 0.01) and BPNS (IGD: 78.4 ± 13.1, HC: 89.4 ± 12.3, *t* = −2.7, *p* = 0.01) were significantly different between individuals with IGD and HCs.

**Table 1:** Demographic and clinical characteristics of individuals with internet gaming disorder (IGD) and healthy control (HC).

	**IGD (*n* = 19)**	**HC (*n* = 20)**	***t***	***p***
Age (years)	23.3 (2.4)	23.4 (1.2)	−0.2	0.6
Education years	15.0 (2.5)	15.4 (1.5).	−0.6	0.5
Intelligence quotient	113.3 (15.6)	108.7 (8.5)	1.1	0.3
Internet addiction test	73.0 (9.7)	24.9 (6.1)	18.4	<0.01
Basic psychological needs scale	78.4 (13.1)	89.4 (12.3)	−2.7	0.01

*Data are given as mean (standard deviation)*.

**Figure 1:**
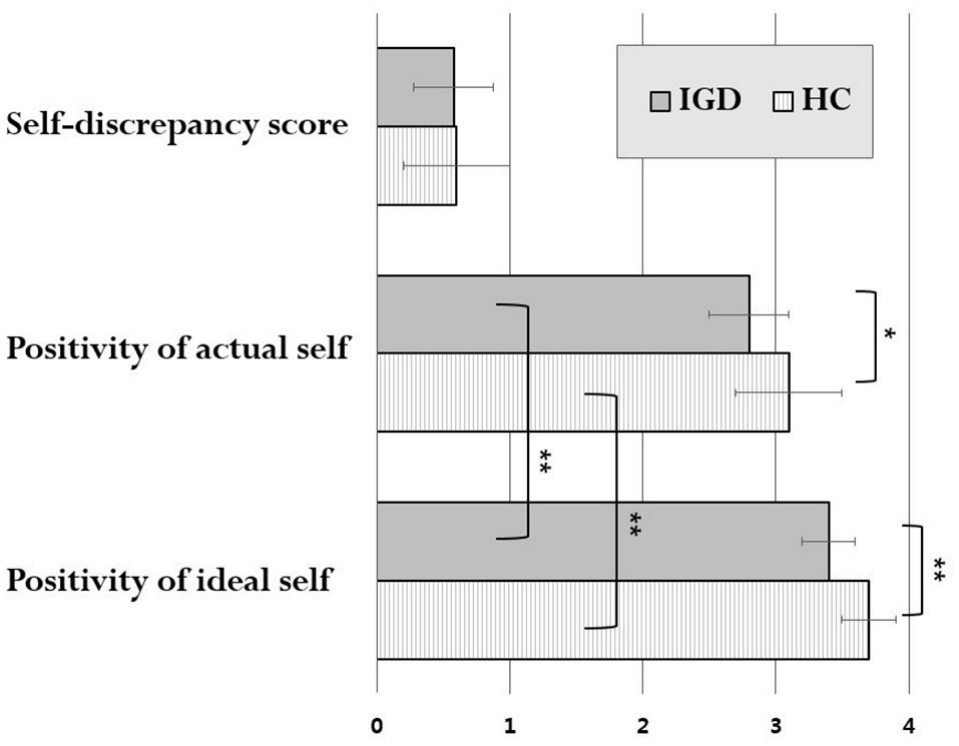
Behavioral responses to the self-concept task. Positivity scores of the ideal self and actual self were significantly lower in individuals with internet gaming disorder (IGD) than in healthy controls (HC). Degree of self-discrepancy (positivity scores of the ideal self—positivity scores of the actual self) was not significantly different between the two groups. *p < 0.05, **p < 0.01.

Figure 1 displays the results of the self-concept task. The main effects of group (*F* = 16.7, *p* < 0.001) and condition (*F* = 69.4, *p* < 0.001) were observed, but no significant group-by-condition interaction effect was found. The positivity scores of the ideal (*t* = −4.6 *p* < 0.01) and actual self (*t* = −2.2, *p* = 0.03) were significantly lower in the IGD group than in the HC group. However, there was no group difference in the self-discrepancy scores (*t* = −0.18, *p* = 0.9). Also, the positivity scores of the ideal self were higher than those of the actual self in both groups (IGD: *t* = 7.9, *p* < 0.01; HC: *t* = 6.4, *p* < 0.01).

The IAT scores were negatively associated with the BPNS scores in individuals with IGD (*r* = −0.52, *p* = 0.02). The self-discrepancy scores were negatively correlated with the BPNS scores (IGD: *r* = −0.8, *p* < 0.01; HC: *r* = −0.5, *p* = 0.01), and these BPNS scores were also correlated with the positivity scores of the actual self in both groups (IGD: *r* = 0.7, *p* < 0.01; HC: *r* = 0.6, *p* < 0.01). There were no statistically significant correlations between the BPNS scores and the positivity scores of the ideal self (IGD: *r* = −0.1, *p* = 0.5; HC: *r* = 0.4, *p* = 0.1).

### Neural response to the self-concept task

**Figure 2:**
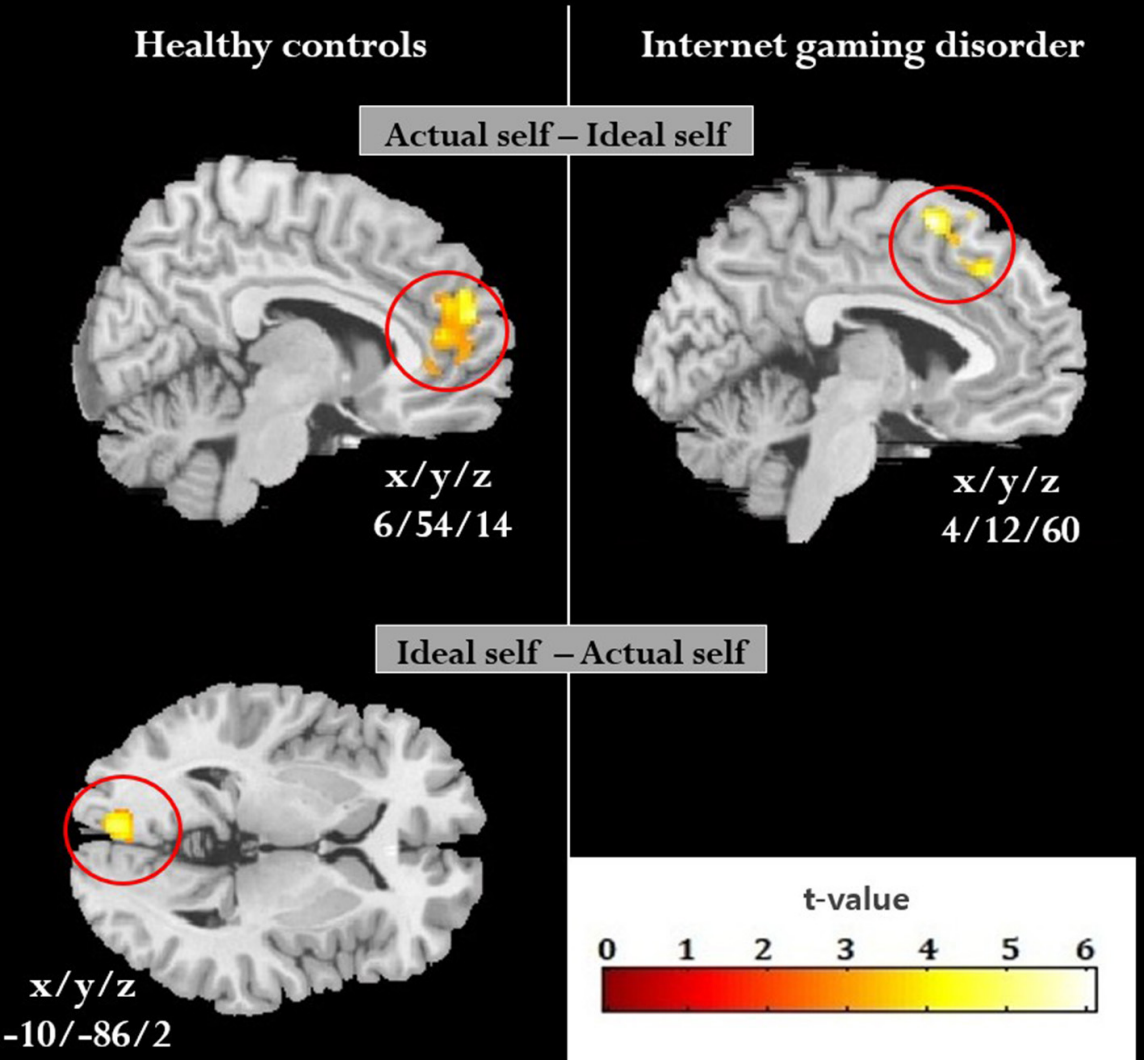
Brain regions showing a significant difference in the comparison between the actual self and ideal self in each group. Increased activity in the actual self compared to the ideal self was found in the bilateral medial prefrontal cortex in healthy controls and the right medial prefrontal cortex in individuals with internet gaming disorder, whereas increased activity in the ideal self compared to the actual self was observed only in the left calcarine cortex in healthy controls.

Figure 2 presents the brain regions related to self-concept in each group. Significantly higher activity in the actual self condition compared to the ideal self condition was observed in the bilateral MPFC (MNI coordinates: 6, 54, 14, voxel number 1,000, *z* = 4.5, *p*_FWE_ < 0.01) in HCs and in the right MPFC (MNI coordinates: 4, 12, 60, voxel number 492, *z* = 4.0, *p*_FWE_ < 0.01) in individuals with IGD. In the ideal self condition compared to the actual self condition, HCs showed significantly higher activity in the left calcarine cortex (MNI coordinates: −10, −86, 2, voxel number 457, *z* = 3.9, *p*_FWE_ = 0.01), whereas individuals with IGD showed no significant result.

**Figure 3:**
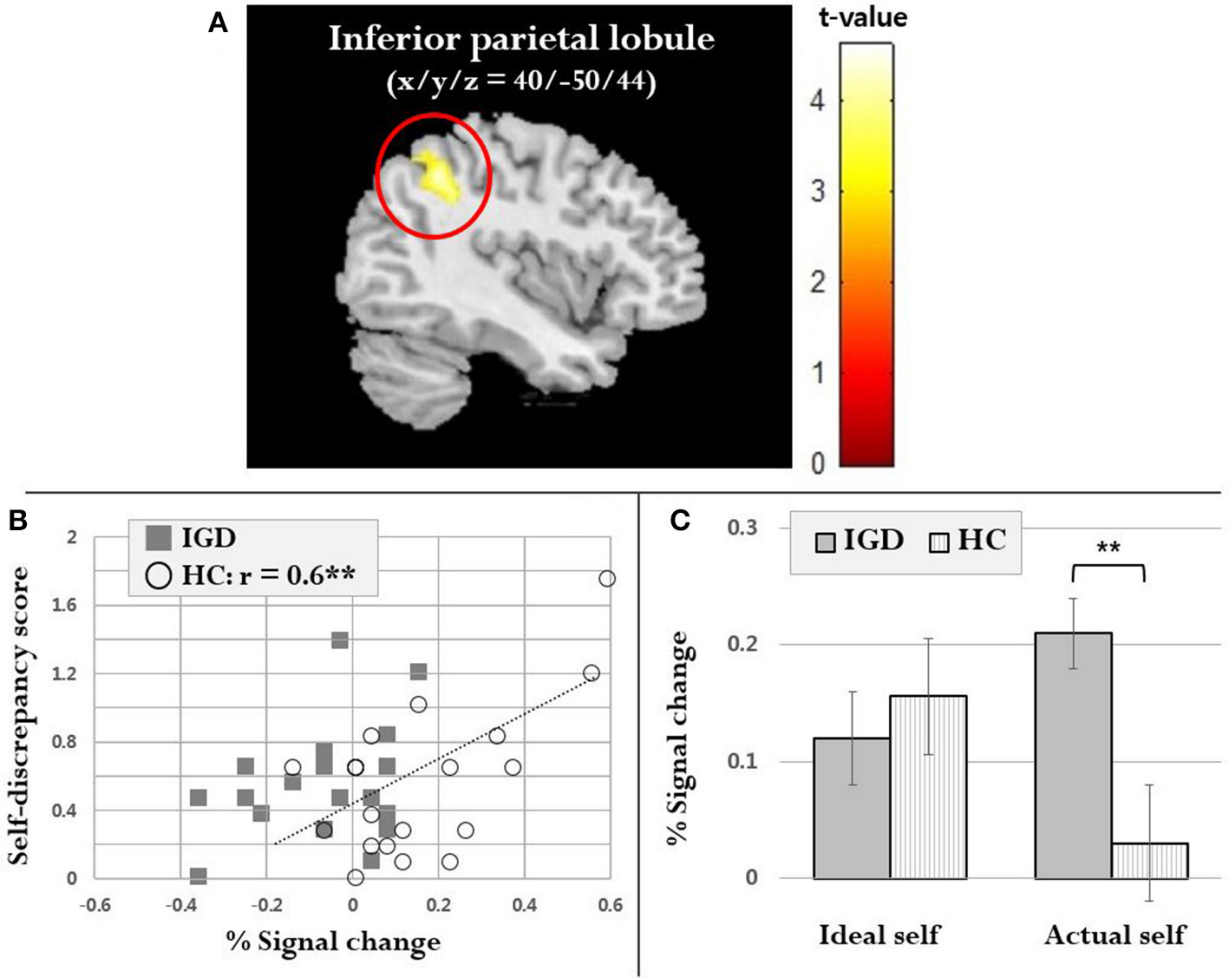
Neural responses during the self-concept task. As shown in **(A)**, individuals with internet gaming disorder (IGD) showed significantly lower inferior parietal lobule (IPL) activity in the self-discrepancy contrast than healthy controls (HC). The correlation between IPL activity in the self-discrepancy contrast and behavioral data is displayed in **(B)**. IPL activity in the ideal self and actual self conditions in each group is displayed in panel **(C)**. **p < 0.01.

Full factorial analysis showed that the main effect of group was observed in the right MPFC (MNI coordinates: 4, 14, 58, voxel number 386, *z* = 4.5, *p*_FWE_ < 0.01) and right caudate (MNI coordinates: 10, 8, 16 voxel number 301, *z* = 3.4, *p*_FWE_ = 0.03), whereas there was no significant main effect of condition and group-by-condition interaction effect. Using two-sample *t*-test on the self-discrepancy contrasts, the right inferior parietal lobule (IPL) showed significantly lower activity in individuals with IGD than in HCs (MNI coordinates 40, −50, 44, voxel number 459, *z* = 4.1, *p*_FWE_ = 0.01) (Figure 3A). IPL activity in the self-discrepancy contrast was positively correlated with the self-discrepancy scores (*r* = 0.6, *p* < 0.01) in HCs, but not in individuals with IGD (Figure 3B). There was no significant correlation between this regional activity and the BPNS scores in both groups (IGD: *r* = −0.2, *p* = 0.3; HC: *r* = −0.1, *p* = 0.7). Meanwhile, IPL activity in the actual self contrast was significantly higher in individuals with IGD than in HCs (*t* = 2.7, *p* < 0.01), whereas no significant group difference was found in the ideal self contrast (Figure 3C).

## Discussion

The purpose of this study was to elucidate the neural correlates of distorted self-concept based on self-discrepancy in individuals with IGD. In individuals with IGD, it was confirmed that they were negatively biased toward their actual self-concept and ideal self-guide rather than HCs. It is a conventional hypothesis that individuals engage in specific actions to reduce self-discrepancy, and similarly individuals with IGD use games as a way to escape negative feelings caused by self-discrepancy (12–14). Self-discrepancy in our patient sample was similar to that in HCs, though self-discrepancy was greater in individuals with IGD vs. HCs in several other studies (12, 14). There are two possibilities for this discrepancy. First, the previous studies involved younger participants than our study. It is important to consider the possibility that self-discrepancy is less likely in older adolescents who have had some degree of self-development than those who had internet addiction since a younger adolescent age. Second, the method of measuring self-discrepancy used in our study might not have been delicate enough to assess the difference. If participants were asked to assess the difference between actual and ideal self-concept directly (12), or if the Likert scale had been expanded as in previous studies (14), a group difference of self-discrepancy might have materialized. In both cases, it does not mean that there was no problem with self-concept in IGD. It should be noted that both actual self-concept and ideal self-guide were negatively biased in individuals with IGD.

Neurobiologically, a meaningful difference was found between individuals with IGD and HCs. For example, the calcarine cortex was more activated when HCs evaluated ideal self-concept compared to actual self-concept. The calcarine cortex is activated in mental imagery processing as well as when actively watching something (38). In the implicit inference process, this area serves as a bridge that enables explicit access when activated. Imagining ideal self-concept would be a more implicit process than speculating actual self-concept and the result could be understood in that sense. On the other hand, the MPFC was more activated in both groups when participants evaluated actual self-concept than when they evaluated ideal self-guide. Given the role of the MPFC in self-referential processing (28, 29), it can be inferred that our task was appropriate for evaluating self-image. In addition, there was a group difference in activity of the MPFC and caudate regardless of the two self conditions. These regions have been known to constitute the reward system and be functionally changed in individuals with IGD (39). Aberrant activation in the MPFC has been understood from the perspective of self-regulation, impulse control, and reward mechanism which are problematic in IGD (30). Hyperactivation in the caudate has been related to habitual craving response in IGD (40).

The main finding of our study is that individuals with IGD showed dysfunctional IPL activity in relation to self-discrepancy. Although the group-by-condition interaction effect was not found, individuals with IGD showed decreased activity in the IPL in the self-discrepancy contrast. As IPL activity was increased in HCs, the self-discrepancy score was also increased. Considering the role of this region as a regulator of negative emotion (41), feeling emotional discomfort might be related to IPL activity in HCs. For individuals with IGD, this kind of protection process might not be operating. Another possibility of the neural difference in self-discrepancy may be due to aberrantly increased activity when evaluating actual self-concept in individuals with IGD. The IPL has been associated with negative valence or arousal (42, 43). In addition, IPL activity is particularly decreased, when dealing with self-related negative words (44). In our study, however, this normal response to reducing IPL activity when dealing with negative words did not occur in individuals with IGD. In this context, the problems of actual self-concept rather than ideal self-guide should be considered more important in individuals with IGD.

A previous longitudinal study has shown a reciprocal relationship; individuals who had low BPNS scores were more likely to become individuals with IGD, and the BPNS scores became lower in individuals with IGD (26). We also confirmed that individuals with IGD were less satisfied with their psychological needs, and the degree of dissatisfaction was associated with the severity of the gaming addiction. In addition, we found that participants with low BPNS scores had problems with their self-image. Participants with lower BPNS scores rated their own discrepancy higher and rated actual self-concept more negatively. It is important to note that the lack of satisfaction with psychological needs was more related to negative actual self-concepts than to ideal self-guide. Because gaming leads to distorted self-concept, individuals with IGD should avoid the positive view that games will enable them to achieve competence, autonomy, and relationships that are not achieved in real life.

Unlike previous tasks that were designed to assess the distance between the actual self and the ideal self in terms of a personality trait, this task was designed to examine the actual self and the ideal self separately. Due to the difference in study design, no activation might be observed in the striatum with regard to self-discrepancy. In addition, a previous study suggested that self-discrepancy primed the desire of good outcome and activated the reward system (27). However, individuals with IGD had negative attitudes of their self-image and dysfunction in processing actual self-concept. Therefore, negative self-related regions might be observed rather than the reward system.

Several limitations should be considered in this study. The major problem was that this study had some recruitment bias for the following reasons. First, to identify IGD-specific neural correlates, we excluded patients who currently had other comorbidities. Second, only male participants in their 20 s were included in this study, and thus it is limited to generalize the result to individuals with IGD in early adolescence or late adulthood. Third, it is difficult to distinguish whether the distorted self was the cause of excessive gaming or the consequences of playing games too much, because of the nature of the cross-sectional study. Fourth, it should be noted that the fMRI task did not evaluate self-discrepancy itself but evaluated it by considering the difference between the actual self and ideal self.

Despite the limitations, our study is meaningful in that the results identify dysfunction in the brain associated with the distorted self in IGD. Individuals with IGD may have problems with emotional regulation or self-evaluation as can be inferred from dysfunction in the IPL. Behaviorally, individuals with IGD had both negative attitude toward actual self-concept and ideal self-guide, though their self-discrepancy was not so great. Negative ideal self-guide in IGD might discourage them from having any goals or motivations to achieve in the future. Special attention should be paid to distorted actual self-concept that has been detected not only behaviorally but also neurobiologically, when understanding the disorder or setting the treatment strategies. Considering the characteristics of the internet gaming environment where users can experience new roles and identities (45), individuals with IGD should pay attention to having distorted self-image.

## Author contributions

All authors listed have made a substantial, direct and intellectual contribution to the work, and approved it for publication.

### Conflict of interest statement

The authors declare that the research was conducted in the absence of any commercial or financial relationships that could be construed as a potential conflict of interest. The reviewer SK and handling Editor declared their shared affiliation at the time of the review.
